# Examining the relationship between maternal mental health-related hospital admissions and childhood developmental vulnerability at school entry in Canada and Australia

**DOI:** 10.1192/bjo.2022.642

**Published:** 2023-01-30

**Authors:** Megan F. Bell, Rebecca Glauert, Leslie L. Roos, Elizabeth Wall-Wieler

**Affiliations:** School of Population and Global Health, University of Western Australia, Western Australia; School of Psychological Science, University of Western Australia, Western Australia; and Telethon Kids Institute, University of Western Australia, Western Australia; School of Population and Global Health, University of Western Australia, Western Australia; and Telethon Kids Institute, University of Western Australia, Western Australia; Department of Community Health Sciences, University of Manitoba, Canada

**Keywords:** Maternal mental illness, epidemiology, in-patient treatment, perinatal psychiatry, child development

## Abstract

**Background:**

It is well established that maternal mental illness is associated with an increased risk of poor development for children. However, inconsistencies in findings regarding the nature of the difficulties children experience may be explained by methodological or geographical differences.

**Aims:**

We used a common methodological approach to compare developmental vulnerability for children whose mothers did and did not have a psychiatric hospital admission between conception and school entry in Manitoba, Canada, and Western Australia, Australia. We aimed to determine if there are common patterns to the type and timing of developmental difficulties across the two settings.

**Method:**

Participants included children who were assessed with the Early Development Instrument in Manitoba, Canada (*n* = 69 785), and Western Australia, Australia (*n* = 19 529). We examined any maternal psychiatric hospital admission (obtained from administrative data) between conception and child's school entry, as well as at specific time points (pregnancy and each year until school entry).

**Results:**

Log-binomial regressions modelled the risk of children of mothers with psychiatric hospital admissions being developmentally vulnerable. In both Manitoba and Western Australia, an increased risk of developmental vulnerability on all domains was found. Children had an increased risk of developmental vulnerability regardless of their age at the time their mother was admitted to hospital.

**Conclusions:**

This cross-national comparison provides further evidence of an increased risk of developmental vulnerability for children whose mothers experience severe mental health difficulties. Provision of preventative services during early childhood to children whose mothers experience mental ill health may help to mitigate developmental difficulties at school entry.

Developmental vulnerability at school entry can have long-lasting effects on children's educational, social and economic outcomes.^1–3^ Children exposed to maternal mental illness in pregnancy and early childhood are at greater risk of experiencing vulnerability regarding cognitive development and psychosocial well-being.^4,5^ Mental illness includes a wide range of disorders with varying degrees of complexity; the type and severity of maternal mental illness can have differing effects on child outcomes.^6^ During pregnancy and the postpartum period, mood and anxiety disorders are most common.^7,8^ The impact of maternal mental illness on child development has been linked to different mechanisms, depending on whether exposure was during pregnancy or after birth. Antenatal mood disorders can affect foetal development as well as behavioural and emotional development well into childhood.^9^ The impact of exposure to postpartum mental illness on childhood development has been attributed more to a child's environment, including insensitive maternal interaction, lack of stimulation at home and social class.^10^ However, inconsistent results across studies could be because of a variety of factors, including differences in study design, policies and access to mental healthcare.^11^

## Measuring and defining maternal mental illness

Maternal mental illness is common, but prevalence estimates vary across studies; a population-based study in the UK^12^ found that the overall prevalence of maternal mental illness was 23.2%, whereas a study in Sweden^13^ found that 5.9% of children were exposed to maternal mental illness. However, these two studies used different measures of maternal mental illness: the UK study defined maternal mental illness by using diagnoses, symptoms and therapy, whereas the Swedish study defined this outcome using diagnoses only. Comparison of these prevalence estimates across countries is therefore not informative, as the measures used to define maternal mental illness are dissimilar. Two recent studies from Canada and Australia used population-level data to examine the relationship between exposure to parental mental illness in early childhood and developmental vulnerability at school entry.^14,15^ Although both studies used similar measures of mental illness (administrative data) and developmental vulnerability at school entry (the Early Development Instrument (EDI)/Australian version of the Early Development Instrument (AvEDI)), these papers had somewhat different findings. Children in the Canadian study showed significantly increased risk of developmental vulnerability on physical health and well-being, social competence or emotional maturity domains if their mother had a depression diagnosis up to the child's fifth birthday. Australian children had significantly increased odds of developmental vulnerability on any of five developmental domains if their mother was admitted to hospital for a psychiatric disorder before the child's school commencement. Differences in findings may be attributed to methodological dissimilarities: the Canadian study looked at maternal depression only, identified from prescriptions and in-patient and ambulatory mental health records, whereas the Australian work examined all maternal and paternal psychiatric diagnoses captured from in-patient records, and did not have access to prescription data. Alternatively, the different findings could be explained by differences in maternal mental health and child development services, access and policies across the two countries.

## Current study

The definition of maternal mental illness has a strong impact on the observed relationship between maternal mental illness and childhood developmental vulnerability. In this study, we use a harmonised approach – defining maternal mental illness as a hospital admission in which the mother had a mental health diagnosis, and by using a common measure of childhood developmental vulnerability – to compare this relationship in Canada and Australia. This will help inform our understanding of how early childhood exposure to maternal mental illness resulting in hospital admission can affect school readiness.

## Method

### Study setting

#### Population profile

The Canadian analysis uses data from the province of Manitoba, which, at the time of the 2016 census, had a population of 1.3 million residents, 55% of whom reside in the capital city of Winnipeg.^16^ The Australian study was conducted in the state of Western Australia, which has a population of 2.6 million, 79% of whom live in the metropolitan capital of Perth.

#### Health services

As in all Canada provinces, Manitoba residents have access to universal healthcare coverage; they do not pay for medical services at point of access. This includes access to prenatal and postpartum care, care for children and access to mental health services. All health services in Canada are provided in the public system. However, service providers and the general public have both voiced significant concerns that current mental health services are not able to meet demands, and access to these services are hindered by long wait times.^17^

Australian health services are provided by a mix of public and private practices. The universal healthcare scheme, Medicare, is available to all Australian citizens and permanent residents, and covers all patient costs in public hospitals. Individuals may also seek mental healthcare services in private hospitals, costs of which may be partially covered by private health insurance. Private health insurance is voluntary in Australia; insurance premiums are primarily paid by the individuals themselves, and occasionally by employers. Private mental health services come with substantial out-of-pocket costs for individuals.^18^ Medicare covers some or all costs of public out-patient services, including mental health services and prenatal and postpartum care; however, a significant proportion of out-patient mental health services are provided by the private sector.^19^ As in Canada, there have long been concerns about barriers to accessing public mental health services, including the capacity of the system to meet demands and long wait times.^19,20^

#### Schools

In Manitoba, the school year runs from September to June. Any child who turns 5 years old by 31 December may attend kindergarten. Although it is not compulsory for children to attend kindergarten in Manitoba, it is offered free of charge in all public schools across the province, with the majority of schools offering half-day programmes.^21^

The school year in Western Australia runs from January to December. The first year of compulsory full-time schooling is the year before grade 1 (i.e. ‘pre-primary’). Children commence pre-primary the year that they will be 5 years old by 30 June.

#### Data

The Manitoba analysis was conducted with linkable administrative data contained in the Manitoba Population Research Data Repository. This repository contains de-identified, routinely collected health and social data linked at the individual level, using an anonymised personal health number for each resident of the province.^22^ Children were linked with information from their mothers by using the Hospital Newborn to Mother Linkage Registry. Linkage methods, confidentiality, privacy and validity have been fully documented.^23,24^ This study joined data from the Manitoba Health Insurance Registry with individual-level information from the Hospital Discharge Abstract Database (DAD) and Manitoba Education.

In Western Australia, anonymised administrative data were merged across the Department of Health and the Commonwealth Department of Education. Datasets were linked by the Western Australia Data Linkage Branch by matching identifiers common to the sets of records (e.g. name, address etc.), using a probabilistic matching approach and clerical review.^25^ Mother–child links within the Family Connections Genealogy System^26^ used information from birth and midwives records. Only de-identified records (with names and addresses removed) were provided to researchers.

### Outcome: early child development

Developmental vulnerability at school entry was assessed with the EDI in Manitoba and the Australian Early Development Census (AEDC), which uses the AvEDI, in Western Australia.^27^ Minor language changes adapted the EDI to an Australian context (e.g. ‘washroom’ was changed to ‘toilet’). In addition, answer options were reduced from a five-point Likert scale to a three-point scale in 18 of the AvEDI items.^27,28^ Both the EDI (a 111-item questionnaire) and AvEDI (a 104-item questionnaire) measure developmental vulnerability across five domains: physical health and well-being, emotional maturity, social competence, language and cognitive skills (school-based), and communication skills and general knowledge.^27^ Supplementary Appendix 1 available at https://doi.org/10.1192/bjo.2022.642 summarises differences in the questions asked in the EDI and AvEDI. The Manitoba measure is assessed by kindergarten teachers once every 2 years in February or March of the academic year; in Western Australia, this measure is assessed by pre-primary teachers once every 3 years in May to July of the academic year.

For each of the five domains, a score (from 0 to 10) is calculated for each child. These scores are analysed at the national level and classified into percentiles. Children scoring in the bottom 10% of a domain are considered ‘developmentally vulnerable’ on that domain.^29^ In addition to examining developmental vulnerability on each of the five domains, we also examined the number of children developmentally vulnerable on at least one domain and children developmentally vulnerable on at least two domains.

### Exposure: maternal mental health-related hospital admissions

The exposure variable in this study was maternal mental health-related hospital admissions, chosen because of the similarities in access and care level across the two settings. Maternal psychiatric hospital admissions in Manitoba were identified from the DAD, provided by Manitoba Health, Seniors and Active Living; and in Western Australia, from the Hospital Morbidity Data Collection (HMDC), provided by the Western Australia Department of Health. The DAD contains information on all Manitoba hospital admissions, and the HMDC contains information on episodes of care for all public and private hospital separations in Western Australia.

Manitoba diagnostic information was recorded with ICD-9-CM^30^ for hospital admissions before 1 April 2004, and ICD-10-CA^31^ for hospital admissions on or after 1 April 2004. Any maternal DAD record during the study period with a primary ICD-9-CM diagnosis code of 290–319 (mental, behavioural and neurodevelopmental disorders) or ICD-10-CA diagnosis code of F00–F99 (mental and behavioural disorders) or O99.3 (mental disorders complicating pregnancy, childbirth and the puerperium), was considered to be a mental health-related hospital admissions.

In Western Australia, diagnostic information was recorded with the ICD-10-AM.^32^ Any maternal HMDC record during the study period with a primary ICD-10-AM code of F00–F99 or O99.3 was considered to be a mental health-related hospital admission.

For each child, the study period started from conception (defined as 9 months before the child's month of birth) up to school entry. Each maternal mental health-related hospital admission was classified according to the age of the child at the date of the mother's separation from hospital. Six non-exclusive groups were created based on the timing of the maternal psychiatric hospital admission: pregnancy, birth to first birthday, first to second birthday, second to third birthday, third to fourth birthday and fourth birthday to school entry. Children could be counted in multiple groups if their mother had a psychiatric hospital admission during multiple time periods.

### Cohort selection

Among Manitoba children enrolled in kindergarten in 2005, 2006, 2009, 2010, 2012, 2014 and 2016 (years in which the EDI was administered) who had information on all five domains of the EDI and completed the EDI after their fourth birthday (*n* = 78 827), we excluded those not born in the province and those who could not be linked to their mothers (*n* = 10 550). The final study sample from Manitoba consisted of 68 274 children (mean age at school entry 5.2 years, s.d. = 0.3).

The Western Australia study population included all children with information on all five domains of the AEDC collected in 2009 (*n* = 23 204). Children were excluded if they were born outside of the state (*n* = 3675). The final study sample consisted of 19 529 children (mean age at school entry 5.1 years, s.d. = 0.3).

### Statistical analyses

Log-binomial regressions estimated the relative risk of developmental vulnerability for children whose mothers have a mental health-related hospital admission compared with children whose mothers do not. This is a descriptive study that aims to describe the burden of developmental vulnerability in children whose mothers have been admitted to hospital with a mental illness diagnosis. For this reason, we present only the unadjusted results. The main analyses included hospital admissions where the primary diagnosis was mental health-related. In both the Manitoba and Western Australia hospital records, mental health-related diagnoses could also appear as additional diagnoses (with a non-mental health-related diagnosis as the primary diagnosis). We therefore conducted a sensitivity analysis to examine the relationship between maternal mental health and child developmental vulnerability by identifying all mothers admitted to hospital during the study period with at least one mental health-related diagnosis recorded in any of the additional diagnosis fields.

All data management, programming and analyses were conducted with SAS version 9.4 for Windows^33^ in both Manitoba and Western Australia.

### Ethics and consent statement

The authors assert that all procedures contributing to this work comply with the ethical standards of the relevant national and institutional committees on human experimentation and with the Helsinki Declaration of 1975, as revised in 2008. All procedures involving human participants in Manitoba were approved by the Health Research Ethics Board at the University of Manitoba (approval number H2019:110) and the Health Information Privacy Commission at Manitoba Health, Seniors and Active Living (approval number HS22691). All procedures involving human participants in Western Australia were approved by the Western Australia Department of Health Human Research Ethics Committee (approval number 2013/65), the University of Western Australia Human Research Ethics Committee (approval number RA/4/1/6651) and the Western Australia Aboriginal Health Ethics Committee (approval number 551). The use of de-identified administrative data did not require informed consent from participants in either Manitoba or Western Australia, as approved by the relevant ethics committees.

## Results

In Manitoba, 1262 (1.8%) children had a mother with a mental health-related hospital admission between conception and school entry. The proportion was larger in Western Australia: 703 (3.6%) children had a mother with a mental health-related hospital admission between conception and school entry. In Manitoba, 27% of children whose mothers had a mental health-related hospital admission were developmentally vulnerable on two or more domains, compared with 16% of children whose mothers did not have a mental health-related hospital admission (Table 1). In Western Australia, the magnitude of the difference was similar: 21% of children whose mothers had a mental health-related hospital admission were developmentally vulnerable on two or more domains, compared with 11% of unexposed children.

**Table 1:** Frequencies and relative risk of developmental vulnerability at school entry by maternal mental health-related hospital admission status

	Manitoba			Western Australia		
	Mother had a mental health-related hospital admission between conception and child entering school			Mother had a mental health-related hospital admission between conception and child entering school		
	Yes (*n* = 1262)	No (*n* = 68 523)		Yes (*n* = 703)	No (*n* = 18 826)	
Developmental domain	Developmentally vulnerable, *n* (%)	Developmentally vulnerable, *n* (%)	Relative risk ratio (95% CI)	Developmentally vulnerable, *n* (%)	Developmentally vulnerable, *n* (%)	Relative risk ratio (95% CI)
2 or more	345 (27.3)	10 937 (16.0)	1.71 (1.56–1.88)	148 (21.0)	2112 (11.2)	1.88 (1.62–2.18)
1 or more	561 (44.5)	19 928 (29.1)	1.53 (1.44–1.63)	269 (38.3)	4362 (23.2)	1.66 (1.50–1.82)
Physical health and well-being	289 (22.9)	9050 (13.2)	1.74 (1.56–1.92)	144 (20.5)	1850 (9.8)	2.09 (1.79–2.43)
Social competence	270 (21.4)	7569 (11.1)	1.94 (1.74–2.16)	83 (11.8)	1354 (7.2)	1.64 (1.33–2.02)
Emotional maturity	275 (21.8)	8583 (12.5)	1.74 (1.56–1.94)	108 (15.4)	1582 (8.4)	1.83 (1.53–2.19)
Language and cognitive skills (school-based)	223 (17.7)	7873 (11.5)	1.54 (1.36–1.74)	145 (20.6)	2088 (11.1)	1.86 (1.60–2.16)
Communication skills and general knowledge	253 (20.1)	9100 (13.3)	1.51 (1.35–1.69)	84 (12.0)	1471 (7.8)	1.53 (1.24–1.88)

### Regression analyses

#### Maternal mental health-related hospital admission

Table 1 displays the frequency, unadjusted relative risk ratios and 95% confidence intervals for children classified as developmentally vulnerable on one or more and two or more domains, and on each of the five developmental domains. Children in both sites whose mothers had a mental health-related hospital admission had an increased risk of being developmentally vulnerable on one or more and two or more domains (Manitoba children, 71% increase and 53% increase, respectively; Western Australia children, 88% and 66% increase, respectively). Manitoba children had a significantly increased risk of developmental vulnerability on all domains if their mother had a mental health-related hospital admission between conception and school entry, with relative risks ranging from 1.51 (communication skills and general knowledge) to 1.94 (social competence). Their Western Australia counterparts also had a significantly increased risk of developmental vulnerability on all five developmental domains, with relative risks ranging from 1.53 (communication skills and general knowledge) to 2.09 (physical health and well-being). In both the Manitoba and Western Australia results, confidence intervals were largely overlapping, indicating the risk was similar for all developmental domains.

#### Age of the child at the time of maternal mental health-related hospital admission

Table 2 displays the frequencies, relative risk ratios and 95% confidence intervals for children classified as developmentally vulnerable on one or more domains relative to the timing of maternal mental health-related hospital admission. At all ages, the risk of developmental vulnerability on at least one domain increased with maternal mental health-related hospital admissions in both jurisdictions. In Manitoba, the largest relative risks were seen for maternal mental health-related hospital admissions during pregnancy and between the child's first and second birthdays. In Western Australia, the largest relative risks were seen for maternal mental health-related hospital admissions during pregnancy, between the child's second and third birthdays, and between the child's fourth birthday and school entry. However, in both jurisdictions, confidence intervals were largely overlapping, indicating a similarity of association for all age groups.

**Table 2:** Frequencies and relative risk of developmental vulnerability at school entry on at least one developmental domain by age of child at time of maternal mental health-related hospital admission

Age of child at time of maternal mental health-related hospital admission	Manitoba			Western Australia		
	Mother had a mental health-related hospital admission between conception and child entering school			Mother had a mental health-related hospital admission between conception and child entering school		
	Yes	No		Yes	No	
	Developmentally vulnerable, *n* (%)	Developmentally vulnerable, *n* (%)	Relative risk ratio (95% CI)	Developmentally vulnerable, *n* (%)	Developmentally vulnerable, *n* (%)	Relative risk ratio (95% CI)
Pregnancy	106 (50.7)	20 383 (29.3)	1.73 (1.51–1.98)	45 (42.9)	4586 (23.6)	1.81 (1.45–2.27)
Birth to first birthday	138 (43.7)	20 351 (29.3)	1.49 (1.31–1.69)	51 (38.6)	4580 (23.6)	1.64 (1.32–2.03)
First to second birthday	129 (53.1)	20 360 (29.3)	1.81 (1.61–2.04)	49 (35.0)	4582 (23.6)	1.48 (1.18–1.86)
Second to third birthday	128 (43.2)	20 361 (29.3)	1.48 (1.29–1.68)	67 (43.2)	4564 (23.6)	1.83 (1.53–2.20)
Third to fourth birthday	120 (44.1)	20 369 (29.3)	1.51 (1.32–1.72)	50 (37.0)	4581 (23.6)	1.57 (1.26–1.96)
Fourth birthday to school entry	128 (43.1)	20 361 (29.3)	1.47 (1.29–1.68)	74 (42.0)	4557 (23.5)	1.79 (1.50–2.13)

#### Sensitivity analyses: any diagnosis

Sensitivity analyses included mothers having any mental health diagnosis recorded during a hospital stay, in addition to those admitted to hospital with a primary mental health-related diagnosis. This approach identified approximately double the number of mothers with mental health diagnoses in both sites (compared with only identifying mothers admitted to hospital with a primary mental health-related diagnosis (Table 3)). In both Manitoba and Western Australia, children whose mothers had a mental health diagnosis recorded during a hospital stay had a significantly increased risk of developmental vulnerability on at least one or two domains. Manitoba children showed the largest risk for developmental vulnerability on the social competence domain (71% increase); in Western Australia, the largest risk ratio was seen for the physical health and well-being domain (100% increase). As with the overall results, overlapping confidence intervals for both the Manitoba and Western Australia results indicated that maternal mental illness (identified from hospital records) had a similar association with all developmental domains.

**Table 3:** Frequencies and relative risk of developmental vulnerability at school entry by maternal mental health-related hospital admission status, any diagnosis

	Manitoba			Western Australia		
	Mother had a mental health-related hospital admission between conception and child entering school			Mother had a mental health-related hospital admission between conception and child entering school		
	Yes (*n* = 2318)	No (*n* = 67 467)		Yes (*n* = 1442)	No (*n* = 18 087)	
Developmental domain	Developmentally vulnerable, *n* (%)	Developmentally vulnerable, *n* (%)	Relative risk ratio (95% CI)	Developmentally vulnerable, *n* (%)	Developmentally vulnerable, *n* (%)	Relative risk ratio (95% CI)
2 or more	963 (41.5)	19 526 (28.9)	1.60 (1.49–1.72)	290 (20.1)	1970 (10.9)	1.85 (1.65–2.06)
1 or more	587 (25.3)	10 695 (15.9)	1.44 (1.37–1.51)	533 (37.0)	4098 (22.7)	1.63 (1.52–1.75)
Physical health and well-being	463 (20.0)	8876 (13.2)	1.52 (1.40–1.65)	274 (19.0)	1720 (9.5)	2.00 (1.78–2.24)
Social competence	434 (18.7)	7405 (11.0)	1.71 (1.56–1.86)	179 (12.4)	1258 (7.0)	1.78 (1.54–2.07)
Emotional maturity	454 (19.6)	8404 (12.5)	1.57 (1.44–1.71)	215 (14.9)	1475 (8.2)	1.83 (1.60–2.09)
Language and cognitive skills (school-based)	409 (17.6)	7687 (11.4)	1.55 (1.41–1.70)	290 (20.1)	1943 (10.7)	1.52 (1.31–1.77)
Communication skills and general knowledge	435 (18.8)	8918 (13.2)	1.42 (1.30–1.55)	168 (11.7)	1387 (7.7)	1.87 (1.68–2.09)

## Discussion

This cross-national comparison of the association between maternal mental health and children's early development found that children had an increased risk of developmental vulnerability at school entry if their mothers had a mental health-related hospital admission at any time between conception and the child entering school. The magnitude of the effect was similar in Manitoba and Western Australia, with risks of developmental vulnerability elevated for all five developmental domains. Even in disparate locations with different healthcare and schooling systems, children exposed to maternal psychiatric illness severe enough to require hospital admission have an increased risk of poorer early development and of starting school ‘not ready’. Approximately 40% of these children were vulnerable on one or more developmental domains, indicating that developmental vulnerability is common in children whose mothers are admitted to hospital for mental illness.

The results of this study are consistent with earlier work from Canada and Australia that used data from the same populations, but slightly different methodological approaches.^14,15^ There are scant other population-level analyses of the association between maternal mental illness and early child development; of those that are available, findings are consistent with the current study. For example, another Australian study using administrative data found that children whose mothers had a mental illness diagnosis after the child's birth had increased odds of developmental vulnerability on each of the five AvEDI domains.^34^ A smaller study from Canada used physician visits and prescription data to identify maternal mood and anxiety disorders, and found strong negative associations with children's social, emotional and physical development.^35^ Our findings also concur with the plethora of literature documenting an association between maternal mental illness and poor child development, using smaller community-based samples.^4,9,36^ The current study advances the literature by using a harmonised approach to demonstrate a strong association between severe maternal mental illness and poor early child development in two separate countries.

In both sites, children had an increased risk of developmental vulnerability on at least one domain if their mothers had a mental health-related hospital admission at any time from pregnancy up until the child's school commencement. The increase in risk was similar for all age groups, indicating that severe maternal mental illness resulting in hospital admission should be considered a risk marker for developmental difficulties in young children regardless of when it occurs. Pre- and postpartum health services for mothers and babies provide opportunities for identifying mothers experiencing mental illness and children at risk of developmental difficulties. In such cases, preventative programmes could be put in place to support families. However, for older children, other avenues are required for identifying those at risk. During the years immediately before school commencement there is generally less surveillance of children by universal maternal and child healthcare services, and children are not in compulsory education. At-risk children may therefore only be identified once the mother presents for psychiatric treatment. Incorporation of the child's needs into treatment planning for the mother is an important consideration.

The sensitivity analysis demonstrated that children whose mother had a mental health diagnosis recorded at the time of hospital admission, and not just those admitted with a primary mental health condition, had a significantly increased risk of developmental vulnerability across all domains. This finding was replicated in both sites, and suggests that a focus only on children whose mothers have been admitted to hospital with a primary mental health difficulty will omit many children whose mothers have experienced mental health difficulties and who may require additional support. In Western Australia, 20% of these children were developmentally vulnerable on two or more domains; in Manitoba, the proportion was more than double, at 41%. Implementation of preventative programmes for children of adults seeking treatment within psychiatric settings will therefore be unlikely to reach all children needing support.

Although twice as many mothers with mental health conditions were identified with this methodology, all mothers with mental health conditions were not captured; we did not include data from ambulatory services, prescriptions or primary care physicians. As such, our comparison groups most likely include children exposed to maternal mental illness. Use of administrative hospital data means that we captured the more severe end of the spectrum of maternal mental health problems. If we were able to identify all mothers with mental health conditions, results could potentially be attenuated because of the inclusion of mothers with less severe psychiatric illness.

Our findings provide further support for the argument that treatment of mental health disorders in adults who have dependent children must consider the family context.^37–39^ Such interventions can help to reduce the burden on children and young people,^40^ and may lead to improvements in children's emotional and behavioural symptoms.^41,42^ However, the current evidence suggests that treatment of maternal mental health difficulties does little to improve developmental difficulties in the child;^5^ additional interventions directly aimed at such children are required. At a minimum, support should involve an assessment of children's needs and referral on to appropriate services, ideally taking place early on in the mother's first presentation to hospital.^43^ Psychoeducation and emotion regulation and skills training are also important components of interventions for children of mothers with mental illness.^40,44^ However, there remain significant training shortfalls and policy, resourcing and system challenges to the implementation of family-focused practice that must be resolved.^40,45^ Furthermore, women may be unwilling to disclose their parenting status because of stigma and/or fear of child protective services involvement.^40,46^ Effective support of both mothers and children within this context requires a sensitive and respectful approach that acknowledges the particular issues associated with parental mental illness.^47^

Strengths of our study include the alignment of outcome and exposure variables in two different countries and the use of population-level data on child development, as reported by teachers, which minimises the risk of recall and reporting bias. Administrative hospital records also enabled the identification of all mothers admitted to hospital with a mental health condition during the study period. However, we did not have any information about onset or duration of maternal mental health difficulties, features which may modify the association with children's developmental outcomes. In addition, although fathers’ mental health data were available in Western Australia, they were not available in Manitoba and therefore not included in the analyses. Previous research has shown an association between paternal mental health difficulties and poorer child development,^14,48^ so future cross-national comparisons should also examine the association between early child development and paternal mental health-related hospital admissions.

This cross-national comparison provides further evidence of an association between maternal mental health difficulties and risks for children's early development in multiple domains. The similar findings in both Australian and Canadian jurisdictions suggest common mechanisms involved in the transmission of risk. All years before school commencement, and not just those immediately postpartum, appear to be an important time for supporting children whose mothers are experiencing mental health difficulties. Such support could mitigate or prevent the development of ongoing social, emotional, cognitive or physical difficulties as children progress through school. Services should be offered to all children whose mothers present with mental health difficulties, and not just those whose mothers have been admitted to hospital with a primary mental health condition.

## Data Availability

The data that support the Canadian findings of this study are available from the Manitoba Centre for Health Policy, but restrictions apply to the availability of these data, which were used with permission for the current study, and so are not publicly available. Data are, however, available from the authors upon reasonable request, and with permission from the University of Manitoba Health Research Ethics Board, the Health Information Privacy Commission at Manitoba Health, Seniors and Active Living, Manitoba Education and Manitoba Families. The data that support the Australian findings are not available for sharing as they are subject to strict security measures to protect the privacy of the individuals whose data are made available for linkage. Other researchers may apply to access the data through the usual ethical and project approval procedures of the Western Australian Data Linkage Branch and the Western Australian Department of Health Human Research Ethics Committee, in addition to approval from relevant data custodians. The authors can provide the parameters for the data application upon request.

## Supplementary material

For supplementary material accompanying this paper visit http://doi.org/10.1192/bjo.2022.642. click here to view supplementary material
