# Supplementary material for: Examining the relationship between maternal mental health-related hospital admissions and childhood developmental vulnerability at school entry in Canada and Australia
Source: BJPsych Open. 2023 Jan 30;9(1):e29. doi: 10.1192/bjo.2022.642 (PMC9970171; doi:10.1192/bjo.2022.642)
Supplement: Supplementary file 1 [file S2056472422006421sup001.docx]

**Appendix 1 – Differences between the EDI and AvEDI**

Based on the Australian version of the Early Development Instrument (AvEDI) checklist and response criteria for 2009 and the Early Development Instrument (EDI) for 2017/2018.

Refer: <https://edi.offordcentre.com/about/what-is-the-edi/> and <https://www.aedc.gov.au/researchers/early-development-instrument>

Questions asked in the AvEDI that are not asked in the EDI:

- How would you rate this child’s daily personal hygiene? (in Physical Wellbeing section)

Questions asked in the EDI that are not asked in the AvEDI:

- Does the child arrive late to school? (in Physical Wellbeing section)
- Does the child show self-confidence? (in Social and Emotional Development section)
- Does the child follow directions? (in Social and Emotional Development section)
- Is the child upset when left by parent/guardian? (in Social and Emotional Development section)
- Does the child fidget? (in Social and Emotional Development section)
- Does the child appear fearful or anxious? (in Social and Emotional Development section)
- Is the child shy? (in Social and Emotional Development section)
- Does the child suck a thumb/finger? (in Social and Emotional Development section)
